# Neoadjuvant chemoradiotherapy improves survival in locally advanced adenocarcinoma of esophagogastric junction compared with neoadjuvant chemotherapy: a propensity score matching analysis

**DOI:** 10.1186/s12893-021-01136-z

**Published:** 2021-03-17

**Authors:** Jing Li, Qun Zhao, Xueke Ge, Yuzhi Song, Yuan Tian, Shuoshuo Wang, Ming Liu, Xueying Qiao

**Affiliations:** 1 Department of Radiation Oncology, The Fourth Hospital of Hebei Medical University, Shijiazhuang, 050011 Hebei China; 2 Department of General Surgery, The Fourth Hospital of Hebei Medical University, Shijiazhuang, 050011 Hebei China

**Keywords:** Adenocarcinoma of the esophagogastric junction, Neoadjuvant treatment, Chemoradiotherapy, Chemotherapy, Propensity score matching analysis

## Abstract

**Background:**

To analyze whether neoadjuvant chemoradiotherapy (nCRT) could improve the survival for patients with adenocarcinoma of the esophagogastric junction compared with neoadjuvant chemotherapy (nCT). Both neoadjuvant chemotherapy alone and chemoradiotherapy before surgery have been shown to improve overall long-term survival for patients with adenocarcinoma in the esophagus or esophagogastric junction compared to surgery alone. It remains controversial whether nCRT is superior to nCT.

**Methods:**

170 Patients with locally advanced (cT3-4NxM0) Siewert II and III adenocarcinoma of the esophagogastric junction (AEG) were treated with neoadjuvant chemotherapy consisting of capecitabine plus oxaliplatin with or without concurrent radiotherapy in the Fourth Hospital of Hebei Medical University. Intensity-modulated radiation therapy (IMRT) was used and delivered in 5 daily fractions of 1.8 Gy per week for 5 weeks (total dose of PTV: 45 Gy). 120 Patients were included in the propensity score matching (PSM) analysis to compare the effects of nCRT with nCT on survival.

**Results:**

With a median follow-up of 41.2 months for patients alive after propensity score matching analysis, the 1- and 3-year OS were 84.8%, 55.0% in nCRT group and 78.3%, 38.3% in nCT group (P = 0.040; HR = 1.65, 95% CI 1.02–2.69). The 1- and 3-year PFS were 84.9%, 49.2% in nCRT group and 68.3%, 29.0% in nCT group (P = 0.010; HR = 1.80, 95% CI 1.14–2.85). The pathological complete response (pCR) was 17.0% in nCRT group and 1.9% in nCT group (P = 0.030). No significant difference was observed in postoperative complications between the two groups.

**Conclusion:**

The nCRT confers a better survival with improved R0 resection rate and pCR rate compared with nCT for the patients with locally advanced AEG.

## Background

Surgical resection remains the main cornerstone of the treatment for resectable advanced adenocarcinoma of the esophagogastric junction (AEG). Neoadjuvant chemotherapy alone (nCT) and chemoradiotherapy (nCRT) have been shown to improve overall long-term survival for patients with adenocarcinoma in the esophagus or esophagogastric junction compared to surgery alone [1–6]. The MAGIC trial showed a significant benefit of perioperative chemotherapy (epirubicin, cisplatin, and infused fluorouracil) plus surgery over surgery alone in R0 resection rates and survival for resectable gastroesophageal cancer [7]. In the CROSS study [3], the long-term follow-up results showed that the neoadjuvant chemoradiotherapy combined with surgery had the overall survival benefits both for squamous cell carcinoma and adenocarcinoma subtypes in patients with resectable esophageal or esophagogastric junctional cancer. So the neoadjuvant treatment has become the standard treatment modality. Neoadjuvant CRT could confer a better local tumor control with improved R0 resection rates, higher complete histological response (pCR) rate, fewer lymph node metastases compared with nCT, but no survival difference was observed between preoperative CRT and CT [8–10]. It remains controversial whether nCRT is superior to nCT. Both nCRT and nCT were undertaken in the clinical practice in our institution. The incidence of Siewert type I tumors is less frequent in Eastern countries than in Western countries. The majority of patients with AEG in Asia have Siewert II and III cancers, which distribution is quite different from western countries. The similar clinicopathologic characteristics were found in AEG II and AEG III, and there was no significant difference in prognosis between AEG II and AEG III [11]. Our previous retrospective study showed that the addition of radiotherapy to preoperative chemotherapy could improve survival with safety, but was not an independent prognostic factor for overall survival (OS) and progression-free survival (PFS) [12]. Only the patients accomplishing preoperative therapy and surgery were enrolled and the intent-to-treat (ITT) method was not used in the study, so bias may exist in evaluating the survival. In this study, all patients who received nCRT/nCT were enrolled and followed up for a longer period in time, and were analyzed by propensity score matching (PSM) analysis to explore whether radiotherapy added to nCT could improve the survival of the patients with locally advanced Siewert II and III AEG.

## Methods

### Eligibility criteria

We reviewed the data of patients with locally advanced (cT3-4NxM0) Siewert II and III AEG and treated by nCRT or nCT in our hospital between March 2012 and December 2015. It was decided by the preference of surgical oncologists and importantly by the preference of the patients if patients received nCT or nCRT. The patients were divided into nCRT group and nCT group. The Review Board of the Fourth Hospital of Hebei Medical University approved this retrospective study (Ethic approval code: 2017MEC004).

### Treatment

Patients were treated with capecitabine plus oxaliplatin for chemotherapy. The chemotherapy comprised intravenous administration of oxaliplatin (130 mg/m^2^) on day 1, followed by orally administration of 1000 mg/m^2^ of capecitabine twice daily for 14 days. The same chemotherapy regimen was given before and after surgery. In the nCRT group, radiotherapy and chemotherapy were performed concurrently. A total radiation dose of 45 Gy by intensity-modulated radiation therapy (IMRT) was administered in 25 fractions of 1.8 Gy, with one time a day and five days per week. The liver, kidneys, spinal cord and heart were organs at risk which were needed to be protected. Surgery was given preferably 6 to 8 weeks after the end of neoadjuvant treatment. Surgical treatment include proximal subtotal gastrectomy/total gastrectomy and extended lymph node dissection (D2 resection). Proximal subtotal gastrectomy and jejunal interposition, proximal subtotal gastrectomy anastomoses with esophagogastric residues, or total gastrectomy anastomoses with Roux-en-Y was conducted through laparotomy.

### Pathologic evaluation

The pathological analysis included complete resection rate and pathological complete response (pCR), which defined as absence of tumor in the final specimen, lymphovascular invasion, nerval invasion, node positive number, node positive rate.

### Follow-up and statistical analysis

The patients were followed up regularly. The follow-up included a complete medical history, a physical examination, chest and abdominal CT or magnetic resonance imaging, or positron emission tomography if possible and endoscopy if clinically indicated. OS was defined the time from the beginning of treatment to the time of death or follow-up deadline. PFS was defined the time from the beginning of treatment to the time of the first tumor progression time or death. The patients were not randomly assigned in our cohort. To minimize the baseline differences between the nCRT group and nCT group, the PSM analysis was conducted with age, sex, Siewert type, Eastern Cooperative Oncology Group score (ECOG), clinical T stage (cT), clinical N stage (cN) and Her-2 status included in the covariates. To evaluate the significance of differences between the two groups, Chi-square test and t-test were used. The OS and PFS were analyzed using Kaplan–Meier method. The log-rank test was used to analyse the differences in survival. P value lower than 0.05 was considered statistically significant. SPSS version 19.0 software was used for all statistical analysis.

## Results

### Patients’ characteristics

**Fig. 1:**
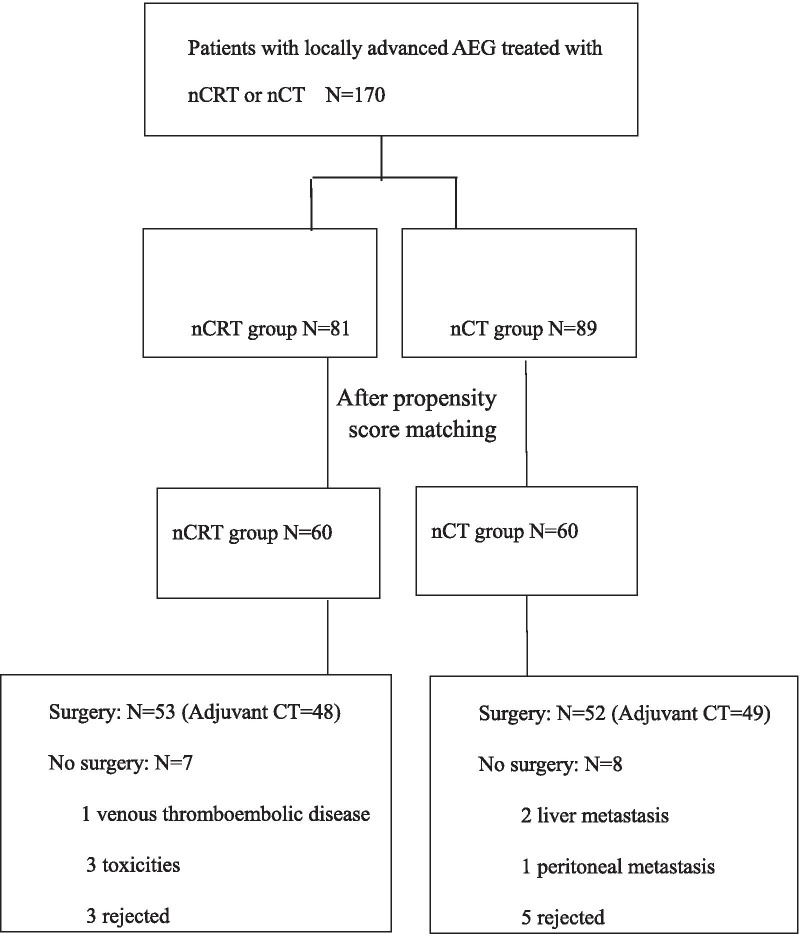
Flow of study enrollment

There were 170 patients with either nCT or nCRT. Eighty-one patients were in nCRT group and eighty-nine patients in nCT groups. The patients’ baseline characteristics were listed in Table 1. After propensity score matching with the covariates which included age, sex, smoking status, Siewert type, ECOG score, cT, cN, and Her-2 status, there were sixty patients in each group, as in Table 1. There were forty-eight and forty-nine patients receiving adjuvant chemotherapy (a median of 4 cycles) respectively in nCRT group and nCT group (P = 0.192). The study enrollment was demonstrated in Fig. 1.

**Table 1:** Patients’ characteristics at the baseline before and after matching

	Before matching			After matching		
	nCRT (%)	nCT (%)	*P*	nCRT (%)	nCT (%)	*P*
All patients	81	89		60	60	
Age			0.273			0.707
≥ 60	49 (60.5)	61 (68.5)		38 (63.3)	36 (60.0)	
< 60	32 (39.5)	28 (31.5)		22 (36.7)	24 (40.0)	
Sex			0.062			0.509
Male	75 (92.6)	74 (83.1)		56 (93.3)	54 (90.0)	
Female	6 (7.4)	15 (16.9)		4 (6.7)	6 (10.0)	
Siewert type			0.000			0.456
II	42 (51.9)	22 (24.7)		26 (43.3)	22 (36.7)	
III	39 (48.1)	67 (75.3)		34 (56.7)	38 (63.3)	
ECOG			0.193			0.729
0	9 (11.1)	5 (5.6)		4 (6.7)	5 (8.3)	
1	72 (88.9)	84 (94.4)		56 (93.3)	55 (91.7)	
cT			0.030			0.822
3	24 (29.6)	14 (15.7)		13 (21.7)	12 (20.0)	
4	57 (70.4)	75 (84.3)		47 (78.3)	48 (80.0)	
cN			0.902			0.500
N0	17 (21.0)	18 (20.2)		14 (23.3)	11 (18.3)	
N+	64 (79.0)	71 (79.8)		46 (76.7)	49 (81.7)	
Her-2			0.258			0.395
Unknown	35 (43.2)	47 (52.8)		26 (43.3)	25 (41.7)	
0/1	28 (34.6)	29 (32.6)		21 (35.0)	24 (40.0)	
2+	15 (18.5)	8 (9.0)		11 (18.3)	6 (10.0)	
3+	3 (3.7)	5 (5.6)		2 (3.3)	5 (8.3)	

*cT* clinical T stage, *cN* clinical N stage

### Survival

**Fig. 2:**
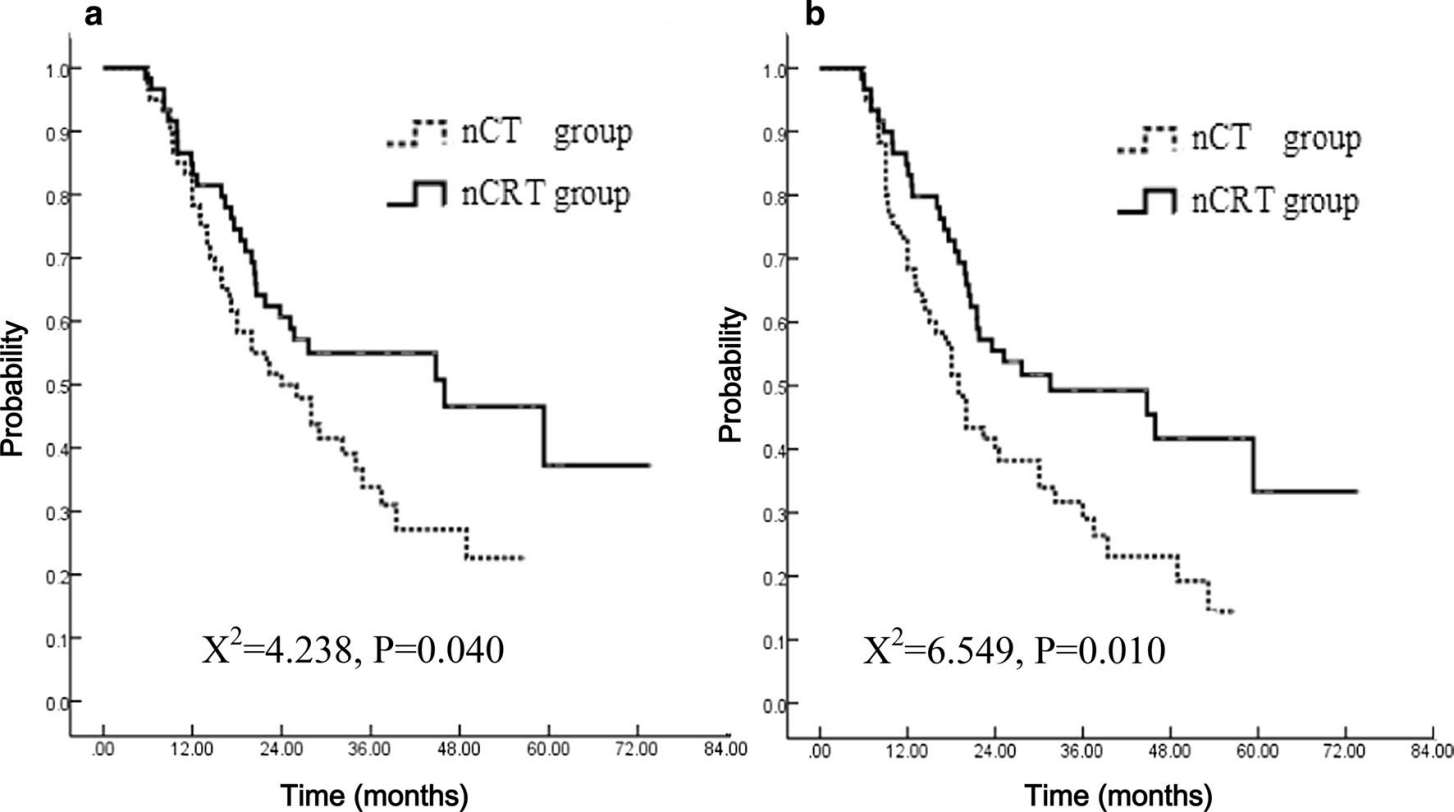
Overall survival (**a**) and Progression-free survival (**b**) according to treatment group (Intent to treat analysis: 60 in nCRT group and 60 in nCT group)

The median follow-up time was 26.0 (4.5–74.2) months for all patients before PSM. The 1- and 3-year OS were 85.1% and 56.7% in nCRT group versus 79.8% and 36.5% in nCT group (p = 0.029, HR = 0.637, 95%CI 0.42–0.96). The 1- and 3-year PFS rates were 83.9% and 52.7% in nCRT group versus 73.0% and 32.1% in nCT group (P = 0.011; HR = 0.61, 95%CI 0.41–0.90). The median follow-up time was 41.2 (22.5–73.7) months for patients alive after PSM. Sixty-nine patients were deceased (29 in nCRT group and 40 in nCT group). The median survival, 1- and 3-year OS were 46.0 months, 84.8% and 55.0% in nCRT group versus 24.0 months, 78.3% and 38.3% in nCT group (p = 0.040, HR = 1.65, 95%CI 1.02–2.69) (Fig. 2a). The median PFS, 1- and 3-year PFS rates were 31.5 months, 84.9% and 49.2% in nCRT group versus 19.0 months, 68.3% and 29.0% in nCT group (p = 0.010; HR = 1.80, 95%CI 1.14–2.85) (Fig. 2b).

Fifty-three patients and fifty-two patients received operation in nCRT group and nCT group respectively. The reasons for receiving no operation were demonstrated in Fig. 1. For the patients with surgery, the median survival, 1- and 3-year OS were 59.3 months, 88.6% and 62.5% in nCRT group versus 28.0 months, 78.8% and 35.3% in nCT group (p = 0.016, HR = 1.94, 95% CI 1.12–3.37). The median PFS, 1- and 3-year PFS rates were 45.9 months, 88.6% and 56.0% in nCRT group versus 19.0 months, 67.3% and 29.9% in nCT group (p = 0.004; HR = 2.09, 95%CI 1.25–3.50).

### Pathologic evaluation after surgery

The median interval time from the end of neoadjuvant therapy to surgery were 45 days (range 40–57) and 47 days (range 43–58) in the nCRT group and nCT group respectively. The median number of examined lymph nodes were 29 (range 9–54) in nCRT group and 32 in nCT group (range 10–72), respectively. R0 resection and pCR were improved in nCRT group compared with nCT group (p = 0.026 and p = 0.030). Lymphovascular invasion, nerval invasion and node positive rate were decreased in nCRT group versus nCT group (p = 0.058, p = 0.011, p < 0.001 respectively) (Table 2).

**Table 2:** Pathological evaluation after surgery in the two groups

	nCRT (n = 60, %)	nCT (n = 60, %)	P
R0	52 (86.7)	43 (71.7)	0.026
R1	1 (1.6)	9 (15.0)	
Non surgery	7 (11.7)	8 (13.3)	

	nCRT (n = 53, %)	nCT (n = 52, %)	P
pCR rate	9 (17.0)	1 (1.9)	0.030
Lymphovascular invasion	5 (9.4)	12 (23.1)	0.058
Nerval invasion	3 (5.7)	12 (23.1)	0.011
Lymph node metastasis	20 (37.7)	30 (57.7)	0.041
Node positive rate	4.4 (55/1245)	23.4 (433/1853)	< 0.001

### Toxicities and postoperative complications

During the neoadjuvant therapy, all patients were assessed weekly about acute toxicities. The toxicities included gastrointestinal toxicities, hematologic toxicities (leukopenia, neutropenia, anemia and thrombocytopenia), pneumonitis and esophagitis. There was no significant difference in gastrointestinal toxicities between nCRT group and nCT group (p = 0.250). There were 7 patients with ≥ grade 3 hematologic toxicities in nCRT group and 0 in nCT group (p < 0.05). And 3 of them did not receive surgery because of the hematological toxicity (thrombocytopenia) and poor general condition thereafter for a long time after chemoradiotherapy. No grade 3 esophagitis and pneumonitis occurred in nCRT group.

Postoperative complications occurred in 20.8% (11/53) of the nCRT patients and 19.2% (10/52) of the nCT patients (p = 0.713). There were 10 patients with pneumonia after surgery in nCRT (one with chylothorax) and 9 patients in nCT group (three with pleural effusion) (Table 3). One patient was with pleural effusion alone in nCRT group and one with venous thromboembolic disease alone in nCT group. In the nCRT group, one patient died of postoperative respiratory failure. There were two patients with intestinal obstruction in nCRT group and one patient in nCT group, who were deceased 17, 18 and 24 months respectively after surgery.

**Table 3:** Postoperative complication in the nCRT group and nCT group

	nCRT group (n = 53,%)	nCT group (n = 52,%)	P
Postoperative complication	11 (20.8)	10 (19.2)	0.713
Pneumonia	10	9	
Pleural effusion	1	0	
Venous thromboembolic disease	0	1	

### Patterns of failure

For patients who were given operation after matching, locoregional recurrence occurred in 3.8% (2/53) patients of nCRT group and 26.9% (14/52) of nCT group (p = 0.001). Distant metastases were observed in 20.8% (11/53) patients of nCRT group and 23.1% (12/52) patients of nCT group (p = 0.774). Locoregional recurrence concurrent with distant metastasis was 3.8% (2/53) in nCRT group and 7.7% (4/52) in nCT group (Table 4). No locoregional recurrence occurred in pCR patients. Only, one pCR patient failed with liver metastasis in nCRT group.

**Table 4:** Patterns of relapse in the nCRT group and nCT group

	nCRT group (n = 53)	nCT group (n = 52)
Locoregional alone	2	14
Distant metastasis alone	11	12
Peritoneal	4	1
Liver	2	7
Lung	2	2
Colon	1	0
Bone	1	1
Liver and bone	1	0
Supraclavicular	0	1
Locoregional with metastasis	2	4
With lung	1	1
With liver	1	1
With peritoneal	0	1
With axillary	0	1

For the 7 patients who received no operation in nCRT group, locoregional recurrence occurred in 2 patients and distant metastases in 4 patients (2 liver metastasis, 1 peritoneal metastasis and 1 peritoneal with liver metastasis). And among the 8 patients in the nCT group, 3 patients developed locoregional recurrence and 2 patients were diagnosed with liver and abdominal metastasis.

## Discussion

The purpose of this study was used ITT method to evaluate whether nCRT could improve the prognosis of the patients with locally advanced AEG compared with nCT. Because the patients were not randomly assigned in our cohort, PSM analysis was used to minimize the statistical bias. In our study, the OS and PFS were both improved in nCRT group compared with nCT group with statistical difference (55.0% and 49.2% versus 38.3% and 29.0% in 3-year OS and PFS) after PSM. The chemoradiotherapy improved 16.7% compared to chemotherapy alone in 3-year OS. The R0 resection rate were higher in nCRT compared with nCT group (98.1% versus 82.7%, P = 0.026) and pCR (17.0% versus 1.9%, P = 0.030). Node positive rate was decreased in the nCRT group compared with nCT group (4.4% versus 23.4%, P < 0.05).

Whether sruvival can benefit from radiotherapy combined with preoperative chemotherapy was still controversial. Sthal et al. [8] compared the preoperative CRT with CT in patients of locally advanced AEG. The results showed that preoperative radiotherapy improved 3-year survival rate by nearly 20% and improved R0 resection rate and pCR. In the randomized studies with pCR as the primary endpoint [9, 10], pCR was improved by nCRT and no significant difference in OS was observed, which favored the patients receiving CRT with no significant difference in surgical toxicity. In a recent systematic review and meta-analysis [13] comparing neoadjuvant CRT with CT for adenocarcinoma of GEJ, no difference was found in terms of median OS, despite a higher pCR rate and a reduced risk of locoregional recurrences for the combined approach. In addition, the propensity-adjusted analysis comparing neoadjuvant chemoradiotherapy and chemotherapy from the National Cancer Data Base (NCDB) of America showed no difference in survival in resectable esophageal and gastroesophageal junction adenocarcinoma, though R0 resection and pCR in the neoadjuvant radiotherapy were improved and lymph node positive rate decreased in neoadjuvant chemoradiotherapy compared with neoadjuvant chemotherapy [14]. There were more patients with smaller tumors and lower proportion of patients with stage III stage in the study with big data base. And we should note that more patients received adjuvant chemotherapy in nCT group than in nCRT group (24.5% versus 7.1%) in the study. In our study, 90.1% and 94.2% patients received adjuvant chemotherapy respectively in nCRT group and nCT group, so the effect of adjuvant chemotherapy on survival was not analyzed. In the aforementioned randomized studies for comparing nCT and nCRT, it is underpowered to analyze the survival difference either because of primary endpoint design [9, 10] or due to the difficulty of accrual [8]. Aforementioned studies included the patients with early stage of AEG and esophageal adenocarcinoma [9, 10, 15], even the patients with squamous cell carcinoma of esophagus [16]. Only Stahl et al. trial studied the patients with AEG in locally advanced stage (T_3-4_N_X_M_0_) [8]. And in our study only the locally advanced (cT3-4NxM0) AEG were included. It may explain the different results in different researches.

In our study, 3 patients were not given operation in nCT group because of disease progression at liver metastasis and peritoneal metastasis. Disease progression in a short period of time may be related to imperfect staging before treatment. Therefore, standard comprehensive staging is essential. Laparoscopy has become an important tool in the diagnosis, staging and treatment of patients with AEG. It was reported that diagnostic staging laparoscopy before treatment could avoid unnecessary laparotomy and let the patients to be able to receive appropriate alternative treatment [17]. Even for the patients with negative pretreatment laparoscopy, post-preoperative treatment laparoscopy may also prevent non-therapeutic laparotomies [18].

From the patterns of failure in our study, nCRT decreased the locoregional failure significantly in nCRT group were 3.8% versus 26.9% in nCT group (p = 0.001) without significant effect on distant metastases compared with nCT (20.8% versus 23.1%) (p = 0.774), which was similar to the result from Stahl M, et al. study [8]. Micrometastases outside the local tumor may affect the long-term result, which means adjuvant chemotherapy or other systemic therapy is important to reduce the risk of distant metastases.

CRT might increase treatment-related death [19, 20]. Common side effects during chemotherapy and chemoradiotherapy were gastrointestinal and hematologic toxicities. In our study, there were 7 patients (11.7%) and 0 with hematologic ≥ grade 3 toxicity during the neoadjuvant treatment in nCRT group and nCT group respectively. In nCRT group, three patients did not undergo surgery due to the hematological toxicities and the poor general condition after chemoradiotherapy. The concurrent chemoradiotherapy may increase the severity of side effects to a certain extent, and make patients loss the opportunity to receive subsequent surgical treatment. But there was no ≥ grade 3 radiation esophagitis or pneumonitis in nCRT group. No significant difference was observed in postoperative complications between the two groups, which was similar in two retrospective studies [21, 22]. Only one patient died of postoperative respiratory failure in nCRT group in 30 days after surgery. The nCRT with capecitabine plus oxaliplatin was well-tolerated. A mata-analysis [23] had confirmed that neoadjuvant CRT plus surgery did not increase the risk of adverse events morbidity.

However, this study had some limitations. As a retrospective study, bias may exist in evaluating the survival even with the propensity score matching analysis. Despite the advantages of nCRT over nCT, some patients may postpone or lose the opportunity to receive surgery or subsequent treatment due to the hematological toxicities. The follow-up time was not long enough to evaluate the 5-year survival precisely. Future randomized studies with big sample sizes are required to prove that adding radiotherapy to preoperative chemotherapy improves prognosis.

## Conclusion

In conclusion, neoadjuvant chemoradiotherapy might improve the overall survival and progression-free survival, increase R0 resection and pCR rate, decrease the locoregional failure over neoadjuvant chemotherapy with no significant increase of postoperative complications for the patients with locally advanced adenocarcinoma of esophagogastric junction.

## Data Availability

The datasets used during the current study are available from the corresponding author on reasonable request.

## Abbreviations

nCRT: Neoadjuvant chemoradiotherapy
nCT: Neoadjuvant chemotherapy
AEG: Adenocarcinoma of the esophagogastric junction
IMRT: Intensity-modulated radiation therapy
PSM: Propensity score matching
pCR: Pathological complete response
OS: Overall survival
PFS: Progression-free survival
ITT: Intent-to-treat
ECOG: Eastern Cooperative Oncology Group
